# Light and elevated temperature induced degradation and recovery of gallium-doped Czochralski-silicon solar cells

**DOI:** 10.1038/s41598-022-11831-3

**Published:** 2022-05-16

**Authors:** Michael Winter, Dominic C. Walter, Byungsul Min, Robby Peibst, Rolf Brendel, Jan Schmidt

**Affiliations:** 1 Institute for Solar Energy Research Hamelin (ISFH), Am Ohrberg 1, 31860 Emmerthal, Germany; 2 Department of Solar Energy, Institute of Solid-State Physics, Leibniz University Hannover, Appelstr. 2, 30167 Hannover, Germany

**Keywords:** Energy science and technology, Materials science, Physics

## Abstract

The fast-firing step commonly applied at the end of solar cell production lines is known to trigger light-induced degradation effects on solar cells made on different silicon materials. In this study, we examine degradation phenomena on high-efficiency solar cells with poly-Si passivating contacts made on Ga-doped Czochralski-grown silicon (Cz-Si) base material under one-sun illumination at elevated temperatures ranging from 80 to 160 °C. The extent of degradation is demonstrated to increase with the applied temperature up to 140 °C. Above 140 °C, the degradation extent decreases with increasing temperature. The degradation of the energy conversion efficiency can be ascribed foremost to a reduction of the short-circuit current and the fill factor and to a lesser extent to a reduction of the open-circuit voltage. The extent of degradation at 140 °C amounts to 0.4%_abs_ of the initial conversion efficiency of 22.1% compared to 0.15%_abs_ at 80 °C. The extent of the efficiency degradation in the examined solar cells is significantly lower (by a factor of ~ 5) compared to solar cells made on B-doped Cz-Si wafers. Importantly, through prolonged illumination at elevated temperatures (e.g. 5 h, 1 sun, 140 °C), an improvement of the conversion efficiency by up to 0.2%_abs_ compared to the initial value is achievable in combination with a permanent regeneration resulting in long-term stable conversion efficiencies above 22%.

## Introduction

Light-induced lifetime degradation effects are frequently observed in many silicon-based materials for solar cell production. The most prominent one is the boron-oxygen (BO) defect activated under illumination in boron-doped Czochralski-grown silicon (Cz-Si)^1^. Another degradation effect is the so called light- and elevated-temperature-induced lifetime degradation (LeTID) first observed on block-cast multicrystalline silicon (mc-Si)^2–4^. Unlike the BO-related degradation, LeTID requires a previous fast-firing step at a high peak temperature^5–7^. LeTID-type effects have been reported in boron-doped Cz-Si^8–10^, in float-zone silicon (FZ-Si)^8,11,12^ and in *n*-type Cz-Si^13^. Gallium is rapidly becoming the dominant dopant for *p*-type silicon solar cell production because of the absence of BO-related lifetime degradation. Several publications showed the significantly more stable performance of Ga-doped solar cells in comparison to B-doped solar cells^1,14–17^. However, there are also more recent studies reporting lifetime instabilities on Ga-doped Cz-Si wafer materials^18–21^, and most recently performance degradation of Ga-doped PERC modules^17^. Our own investigations showed that the temperature, at which the light-induced degradation is performed, has a strong impact on the extent of bulk lifetime degradation in Ga-doped Cz-Si^20^. With increasing temperature the extent of degradation is also increasing. In our recent study^20^, we demonstrated e.g. that illumination of 1 Ω cm Ga-doped Cz-Si wafers performed at 140 °C may result in carrier lifetimes in the fully degraded state τ_d_ of around ~ 500 µs (initial lifetime τ_0_ was ~ 2300 µs). Compared to degradation performed at 90 °C (τ_d_ = 1300 µs), this corresponds to an increase of the corresponding effective defect concentration ${N}_{\text{d}}^{*}=1/{\tau }_{\text{d}}-1/{\tau }_{0}$ by a factor of 5. The reported lifetimes were measured at an excess carrier concentration of Δ*n* = 10^15^ cm^−3^. Another interesting observation was that the illumination intensity used for defect activation can have an impact on the degradation extent^19,20^.

In the present study, we examine, whether the light- and elevated-temperature-induced lifetime degradation (LeTID) observed on Ga-doped Cz-Si wafers is also the cause of the existing but small instabilities observed in solar cells made on Ga-doped Cz-Si wafers. We fabricated POLy-Si on Oxide (POLO) back-junction (BJ) solar cells^22,23^ and perform degradation experiments at an illumination intensity of 1 sun at temperatures ranging from 80 to 160 °C.

## Experimental details

**Figure 1:**
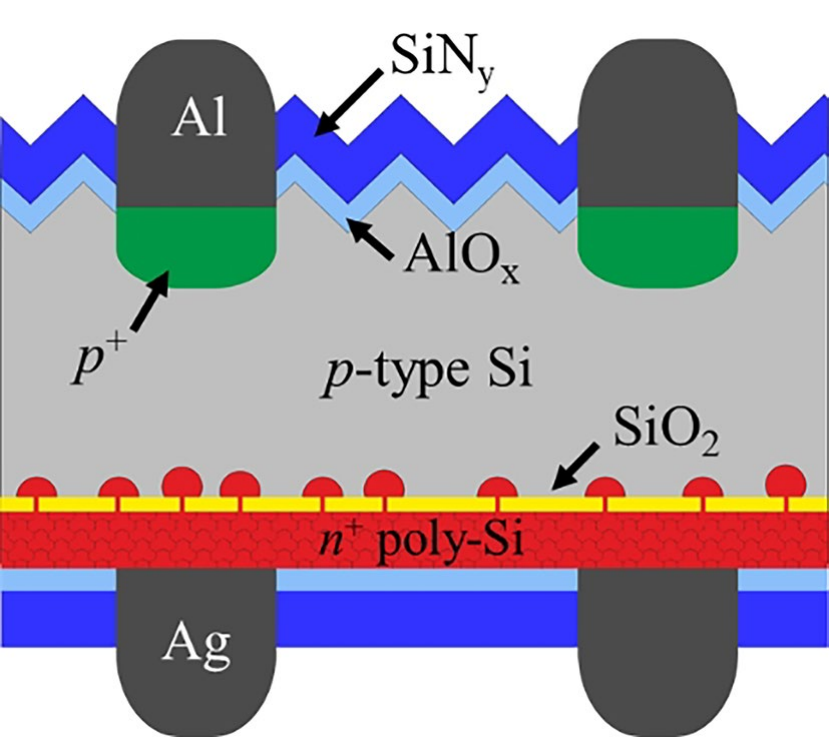
Schematic of the POLO back-junction solar cell^22,23^.

Figure 1 shows the cell structure of a POLO back junction cell. Details can be found elsewhere^23^. The process sequence starts with an industrial gallium-doped *p*-type Cz-Si wafer (M2 size) with a resistivity of 0.8 Ω cm. An (according to ellipsometry measurements) 1.6 nm-thick interfacial oxide layer grows wet-chemically in de-ionized water with diluted ozone, and is then capped by 200 nm of an in-situ phosphorous-doped poly-Si layer deposited in an LPCVD furnace. During a high-temperature step at 840 °C in a quartz-tube furnace, the interfacial oxide layer breaks up and forms the POLO contact. Simultaneously, 150 nm-thick oxide layers grow on poly-Si layers on both surfaces. The oxide layer on the cell front side is then completely removed. A subsequent KOH-based solution textures the front side, while the poly-Si layer at the cell rear side is protected by the thick oxide layer. We then deposit a stack consisting of 10 nm aluminum oxide (AlO_*x*_) and 80 nm or 60 nm silicon nitride (SiN_*y*_) with a refractive index of *n* = 2.05 on the cell front and rear sides, respectively. At the cell front, we locally open the dielectric stack by a laser to form contact openings. The contacts are realized by aluminum-paste screen printing at the front side and silver-paste screen printing at the rear side followed by a co-firing process at a set-peak temperature of 810 °C and a band velocity of 6 m/min in an industrial conveyor belt furnace.

After processing, the finished solar cells are illuminated by 1 sun light intensity (≙ 100 mW cm^−2^) using a halogen lamp on a hotplate at elevated temperatures between 80 and 160 °C. We measure the illumination intensity with a calibrated reference silicon solar cell. The lateral variation of the illumination intensity is less than ± 0.1 suns, whereas the temperature during the illumination time is constant within a range of ± 2 °C. Depending on the experiment, the cells are either (1) illuminated at the above conditions for up to 1400 h (long-term stability test) or (2) illuminated at a reduced intensity of 0.5 suns for ~ 15 min, resulting in a recovery of the cell parameters after degradation. The cell temperature during this low-temperature illumination step is (44 ± 4) °C, as induced by the radiative heating of the halogen lamps.

The *IV* measurements are performed under AM1.5G illumination at 25 °C using a LOANA system from pvtools.

## Results and discussion

### Long-term stability test

**Figure 2:**
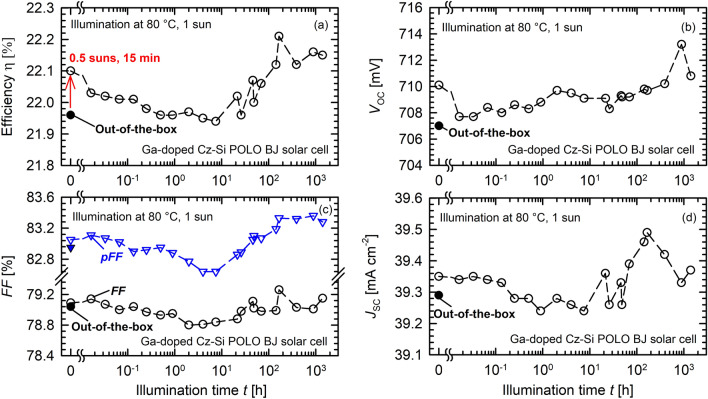
Long-term one-sun illumination of a POLO BJ solar cell on Ga-doped Cz-Si at 80 °C for 1400 h. Shown are the measured parameters (**a**) energy conversion efficiency η, (**b**) open-circuit voltage *V*_OC_, (**c**) fill factor *FF* together with the pseudo fill factor *pFF*, and (**d**) short-circuit current density *J*_SC_ versus the illumination time *t* (open circles). In addition, the initial values measured directly after contact firing are shown (closed symbols). Before the experiment starts, the solar cell is illuminated at 0.5 suns for 15 min, increasing the efficiency from its initial value (slightly below 22%) to 22.1%.

Figure 2 shows the evolution of the measured cell parameters energy conversion efficiency η (Fig. 2a), open-circuit voltage *V*_OC_ (Fig. 2b), fill factor *FF* together with the pseudo fill factor *pFF* (Fig. 2c), and the short-circuit current density *J*_SC_ (Fig. 2d) under one-sun illumination at 80 °C for up to 1400 h. The efficiency η decreases from 22.10 to 21.95% within 10 h of one-sun illumination (Fig. 2a), before a recovery of the efficiency up above the initial efficiency of 22.10% is observed. The degradation extent amounts to ~ 0.7% relative. Although some degradation is observable, the extent of degradation seems to be very small, in particular when compared to solar cells made on B-doped Cz-Si wafers, where degradations extents of up to 10%_rel_ have been reported^1^. The small LeTID effect visible in Fig. 2a has its cause in all three parameters shown in Fig. 2b–d, which are *V*_OC_, *FF*, and *J*_SC_. The extent of degradation, however, is only slightly larger than the initial improvement under illumination at 0.5 suns at ~ 44 °C for 15 min before increasing the temperature. Whether this is because an additional light-induced defect is activated (e.g., a small contamination with iron) or the defect state after processing is already activated and is deactivated again during low-temperature illumination remains to be clarified, though lifetime experiments indicate the first option is more likely.

The short-circuit current density of BJ solar cells is quite sensitive to the bulk lifetime. Minority charge-carriers generated primarily at the front side of the BJ solar cell have to diffuse to the rear side. A more pronounced decrease in *J*_SC_ is therefore expected for the activation of a bulk defect in back-junction solar cells compared to a conventional front-junction cell. The observed decrease in *FF* (Fig. 2c, black circles) could be due to the activation of a bulk defect resulting in a pronounced injection dependence of the bulk carrier lifetime^24^. To exclude any influence of the series and contact resistances, the pseudo fill factor *pFF* is also shown in Fig. 2c (blue triangles). The *pFF* is extracted using the *J*_SC_-*V*_OC_ measurement implemented into the LOANA tool^25,26^. Indeed, less scattering of the data is visible while the general evolution of *FF* and *pFF* are the same. We have verified on reference samples that no degradation of the surface passivation occurs during the illuminated annealing treatments due to the relatively low firing temperature applied. Besides, the time constants of surface-related degradation is significantly larger than all time constants observed in the present study^27,28^.

### Temperature-dependent degradation

**Figure 3:**
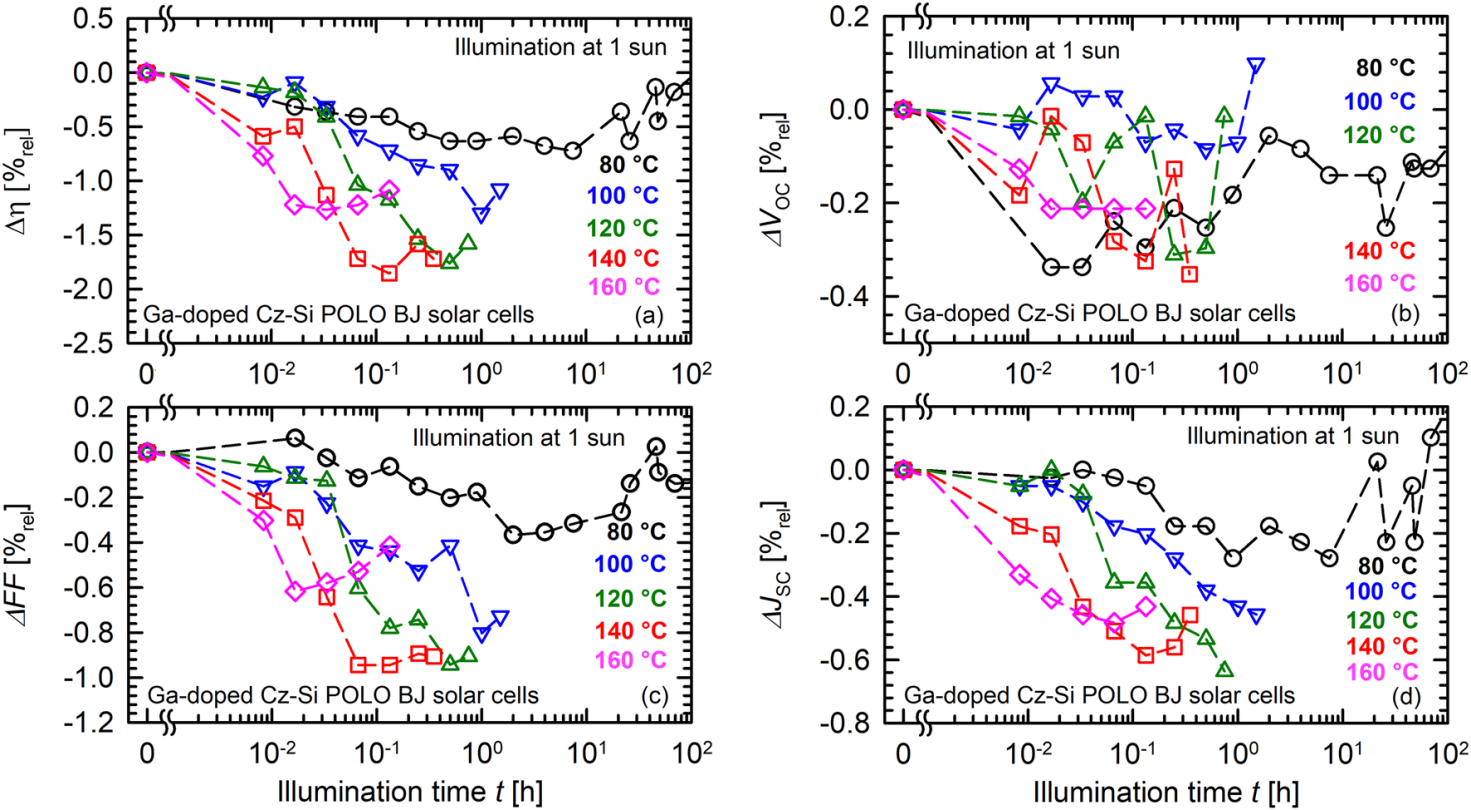
Temperature-dependent light-induced degradation of Ga-doped Cz-Si POLO BJ solar cells at 1 sun light intensity and temperatures ranging from 80 to 160 °C. Shown are the relative changes in the measured cell parameters (**a**) energy conversion efficiency η, (**b**) open-circuit voltage *V*_OC_, (**c**) fill factor *FF*, and (**d**) short-circuit current density *J*_SC_ versus the illumination time *t*.

Figure 3 shows a temperature variation between 80 and 160 °C at an illumination intensity of 1 sun. To make comparisons easier, the relative changes of the four cell parameters are depicted instead of the absolute values. Moreover, the data at 80 °C is the same as shown in Fig. 2. The original data can be found in the supplementary information in Supplementary Fig. S1. Here, for the sake of clarity, we will limit the discussion to the relative data shown in Fig. 3. Figure 3a clearly shows that there is a dependence of the degradation extent on the temperature. Whereas at 80 °C the maximum decrease in conversion efficiency is only 0.15%_abs_, the decrease at 140 °C is 0.4%_abs_. This corresponds to an increase in the relative degradation extent from 0.7%_rel_ to 2%_rel_ as shown in Fig. 3a. The maximum extent of relative degradation increases approximately linearly with increasing temperature between 80 and 140 °C. For temperatures larger than 140 °C, the relative degradation decreases again, as can be seen in Fig. 3a (pink diamonds).

**Figure 4:**
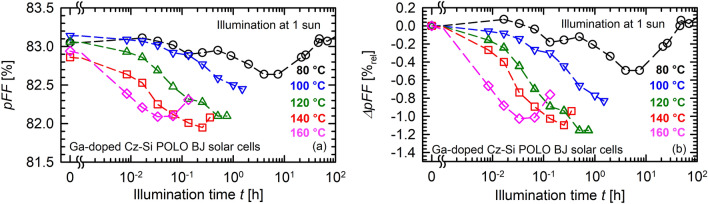
Temperature-dependent light-induced degradation of the pseudo fill factor *pFF* of Ga-doped Cz-Si POLO BJ solar cells at 1 sun light intensity and temperatures ranging from 80 to 160 °C. Shown are the absolute changes (**a**) and the relative changes (**b**) versus the illumination time *t*.

The cause of the degradation can be found in all three parameters shown in the Fig. 3b–d. However, the pronounced temperature dependence is predominantly caused by the fill factor and the short-circuit current density changes. Whereas *FF* degrades by 0.4%_rel_ at 80 °C, this maximum degradation increases to 1%_rel_ at 140 °C. *J*_SC_ is reduced by 0.3%_rel_ in the degraded state at 80 °C compared to its initial value. This degradation increases to 0.6%_rel_ at 140 °C. Due to relatively small *V*_OC_ changes in comparison with the scattering of the measurement data, a clear dependence of the *V*_OC_ degradation on temperature is not seen in the data. To exclude any influence of series and contact resistances on the temperature-dependent degradation of the fill factor in Fig. 3c, the pseudo fill factor *pFF* is shown in Fig. 4a. In Fig. 4b the relative changes are shown analogously to the representation in Fig. 3c. Both Fig. 4a,b confirm the independence of the observed degradation on the series resistance. The degradation extent of *pFF* increases from 0.5%_rel_ at 80 °C to 1.2%_rel_ at 120 °C, before a saturation (140 °C) followed by a decrease (160 °C) of the maximum degradation extent can be observed in Fig. 4b. Note that the degradation data recorded in the temperature range 100–160 °C in Fig. 3 were actually all measured on the same solar cell.

After complete one-sun degradation at different temperatures (80–160 °C), the cells are illuminated at a lower intensity of 0.5 suns, which corresponds to an illumination temperature of ~ 44 °C without active heating of the cells. All four parameters η, *V*_OC_, *FF*, and *J*_SC_ increase again and reach values comparable with the ones before degradation. Afterwards, a degradation at elevated temperature is again possible. The (temporary) recovery takes about 15–30 min at 0.5 suns and ~ 44 °C. Details can be found in our recent publication^20^.

### Lifetime reference

**Figure 5:**
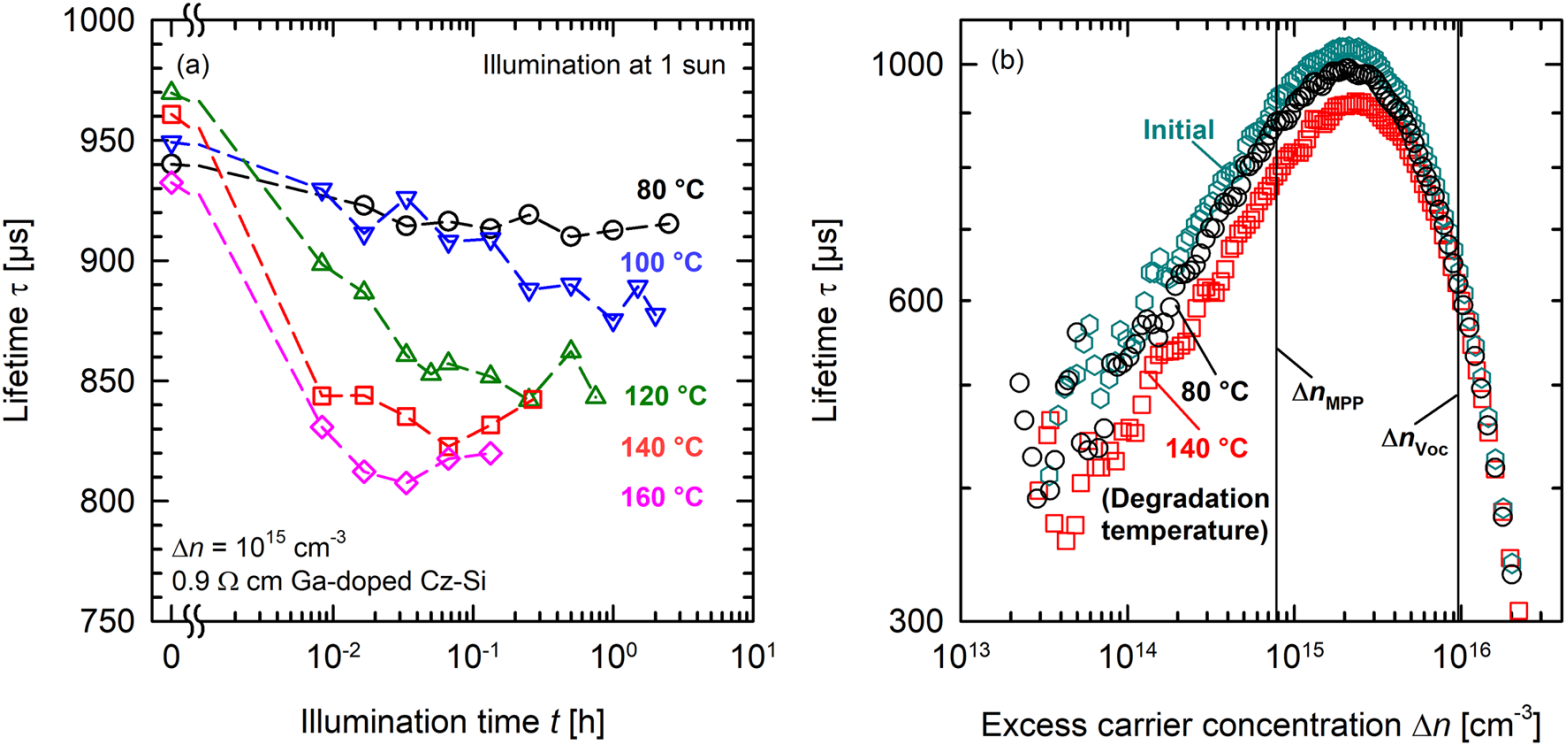
Lifetime degradation of a Ga-doped Cz-Si sample (cell without metallisation) during one-sun illumination. Shown is (**a**) the lifetime degradation at temperatures ranging from 80 to 160 °C at one-sun illumination and (**b**) the injection-dependent lifetimes before degradation (initial, cyan hexagons) and after degradation for 30 min at 80 °C (black circles) and 4 min at 140 °C (red squares).

The measurements on solar cells show very similar behaviour as our lifetime measurements performed on Ga-doped Cz-Si wafers, which is a clear indication that the activation of a bulk defect is responsible for the observed LeTID effect. Figure 5 shows the corresponding lifetime measurements performed on a Ga-doped Cz-Si wafer with passivated surfaces (one-sun illumination at 80 to 160 °C). The sample corresponds to a solar cell without metallisation on the front and rear side. The lifetimes are measured at ~ 30 °C using a WCT-120 lifetime tester from Sinton Instruments. Carrier lifetimes measured at an excess carrier concentration of Δ*n* = 10^15^ cm^−3^ are shown in Fig. 5a. After each degradation step, the defect is deactivated at 0.5 suns at 44 °C before increasing the temperature for the next degradation cycle. The temperature dependence of the degradation extent is clearly visible. Although the defect concentration still increases by a factor of ~ 5 between degradation at 80 °C and 140 °C, the extent of degradation shown in Fig. 5 is lower than what we measured in our previous study^20^. The overall defect concentration in our present study is lower by a factor of 10. There are several possible explanations for this. Both, the set-peak temperature (i.e., 810 °C) and the band velocity (6 m min^−1^) of the firing furnace were lower in the present study, than in our previous study (850 °C, 6.8 m min^−1^). Whereas the lower firing peak temperature would result in less hydrogen diffusing into the silicon bulk, a slower cooling ramp has also been shown to have significant impact on the extent of degradation^29^. Another difference is the sample structure with poly-Si on the back of our lifetime sample instead of a symmetrical stack of AlO_*x*_ and SiN_*y*_. To exactly reveal the causes for the apparent discrepancy between the present and our previous study, more specific experiments are required.

Figure 5b shows the injection-dependent lifetimes before degradation (initial, cyan hexagons) and after degradation for 30 min at 80 °C (black circles) and 4 min at 140 °C (red squares) for our reference lifetime sample. In addition, the approximate excess carrier concentrations are shown for the maximum power point (MPP) Δ*n*_MPP_ and under open-circuit conditions Δ*n*_*V*oc_ (black lines). Whereas the difference between the degradation at 80 °C and 140 °C is clearly visible at Δ*n*_MPP_, it is almost non-existent at the much higher Δ*n*_*V*oc_. This could explain why the temperature dependence of the defect activation is clearly visible in *FF* (Fig. 3c) and *pFF* (Fig. 4), whereas *V*_OC_ shows mainly scattering (Fig. 3b).

### Permanent defect deactivation and absolute improvement

**Figure 6:**
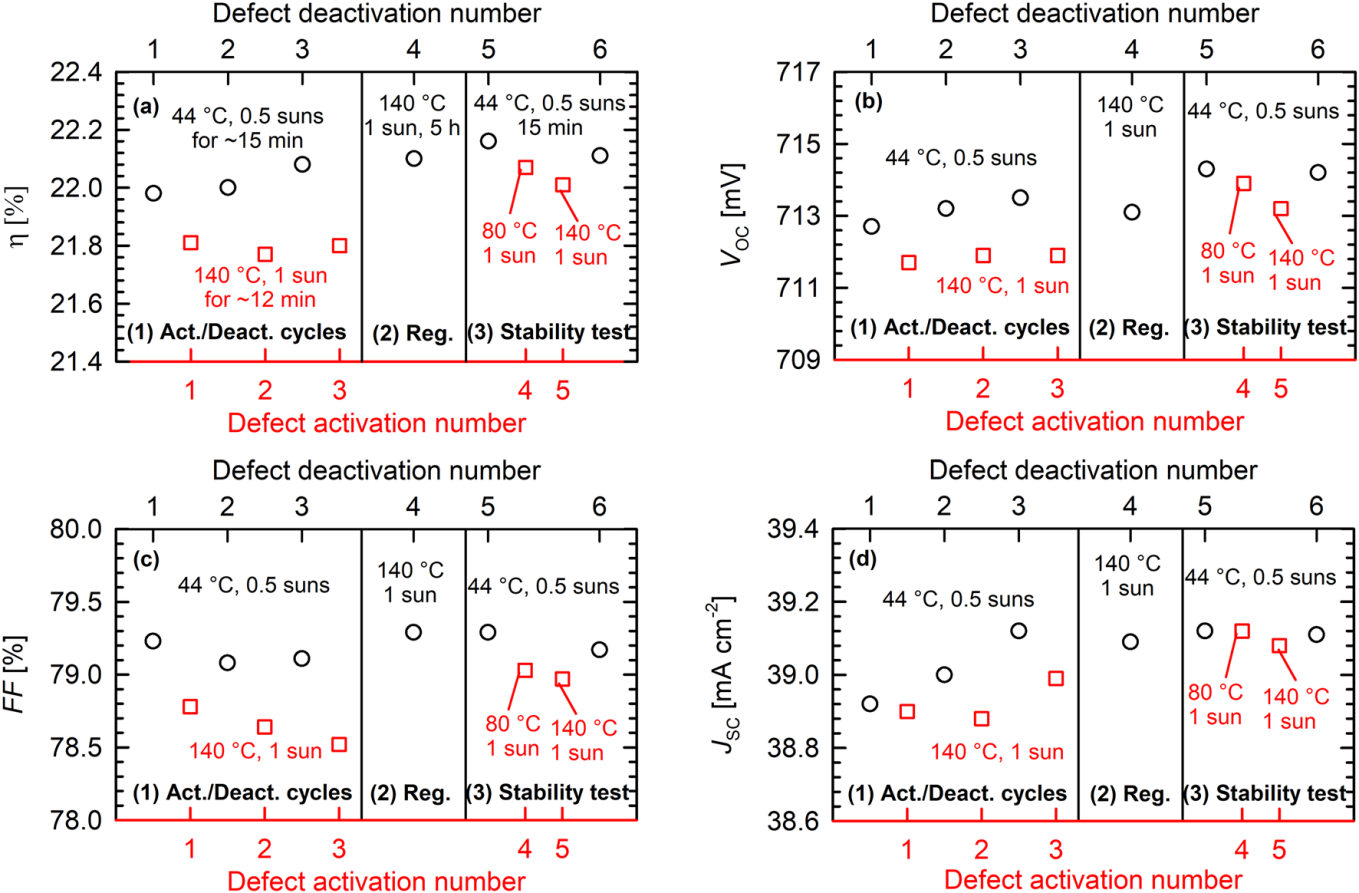
Reversibility of a Ga-doped Cz-Si POLO BJ solar cell through consecutive activation (140 °C, 1 sun, 12 min)/deactivation (44 °C, 0.5 suns, 15 min) cycles (1), regeneration through prolonged illumination at elevated temperatures (140 °C, 1 sun, 5 h) (2), and test of the stability of the regeneration at 80 and 140 °C (3). Shown are the measured cell parameters (**a**) energy conversion efficiency η, (**b**) open-circuit voltage *V*_OC_, (**c**) fill factor *FF*, and (**d**) short-circuit current density *J*_SC_.

A long-term-stable improvement of the cell performance by up to 0.2%_abs_ is possible by prolonged illumination at elevated temperatures (e.g., at 140 °C and 1 sun for 5 h). Figure 6 shows several consecutive defect activation/deactivation cycles on the same solar cell. For the sake of clarity and unlike in Figs. 2 and 3, in Fig. 6 only the respective highest and lowest values of each cycle are shown instead of the full degradation curves. The x-axes do show the progress of the experiment from the left to the right by numbering the respective deactivation and activation step consecutively. The respective degradation curves (numbered from 1 to 5 according to the numbering of the activation step are shown as supplementary information in Supplementary Fig. S2. But we will limit the discussion to Fig. 6. Each of the four subplots (a–d) in Fig. 6 for η, *V*_OC_, *FF*, and *J*_SC_ is separated into 3 stages (1–3). The first stage (1) presents three consecutive cycles of defect deactivation (black circles, upper axis) at ~ 44 °C and 0.5 suns, and defect activation (red squares, lower axis) at 140 °C and 1 sun. It shows the reversibility of the degradation effect under constant defect activation conditions. Please note, however, that the decreasing trend of the degraded *FF* in Fig. 6c(1) is not significant. The reason is simply that we chose to display the respective lowest values of the conversion efficiency without taking the scattering in the *FF* measurement into account. Stage (2) of each subplot (a–d) in Fig. 6 shows the state after a prolonged illumination at 140 °C and 1 sun for 5 h. According to our previous results on Ga-doped Cz-Si lifetime samples, this treatment results in a permanent defect deactivation which corresponds to a permanent regeneration of the bulk lifetime^20^. The conversion efficiency η in Fig. 6a(2) reaches a value of 22.10%. To test the stability of the regenerated solar cell, we apply in stage (3) another defect deactivation/activation cycle, starting with the low-temperature illumination at 0.5 suns and 44 °C (deactivation). The conversion efficiency η improves further to 22.16%, which is an improvement of 0.2%_abs_ compared to the initial η of 21.96% at the very beginning of this experiment. This general improvement is visible in the black circles in Fig. 6a and can be attributed to an increase of both *V*_OC_ (Fig. 6b) and *J*_SC_ (Fig. 6d). A similar improvement of the cell performance has very recently been reported on Ga-doped Cz-Si PERC solar cells by means of a two-step bias treatment^30^.

Subsequently, in stage (3) of Fig. 6, the stability of the regenerated cell is tested in a two-step increase of the temperature, first to 80 °C, then to 140 °C at a constant illumination intensity of 1 sun (red squares). A significantly reduced degradation of only 0.15%_abs_ after illumination at 140 °C and 1 suns is now observed, which is halved compared to the degradation before the regeneration step was applied. Even after maximum degradation at 140 °C an efficiency of 22.0% is obtained. The regeneration treatment can hence be regarded as highly effective, as reported in our recent lifetime study^20^.

## Conclusions

In this contribution, we have performed illumination experiments at elevated temperatures between 80 and 160 °C and 1 sun halogen lamp intensity on POLO BJ cells with a Ga-doped Cz-Si base. POLO BJ cells are relatively stable regarding light-induced degradation which we have demonstrated through long-term illumination at 80 °C for 1400 h. We observed, however, a slight degradation of the cell performance, the extent of which increases with the temperature. Whereas at 80 °C the degradation in energy conversion efficiency is 0.15%_abs_, the degradation extent is increased to 0.4%_abs_ under illumination at 140 °C. This corresponds to an increase in the relative degradation extent from 0.7%_rel_ at 80 °C to 2%_rel_ at 140 °C. For temperatures larger than 140 °C, the degradation extent decreases again. Through illumination at low temperatures and illumination intensities (i.e., at 44 °C and 0.5 suns)—quite common conditions during operation in the field—the degradation effect can be temporarily reversed. Interestingly, the temperature-dependent degradation of the energy conversion efficiency can be foremost ascribed to a reduction of the short-circuit current density and the fill factor and to a lesser extent to a reduction of the open-circuit voltage. This could be explained by the injection dependence of the lifetime of the activated defect, resulting in a more severe degradation of the lifetime for excess carrier concentrations at the maximum-power-point than under open-circuit conditions. Both lifetime measurements on a lifetime sample processed in parallel to the solar cells (corresponding to a cell without metallisation on the front and rear sides) and our previous study^20^ clearly indicate that the activation of a bulk defect is responsible for the observed LeTID effect on the Ga-doped Cz-Si solar cells. In our recent lifetime study^20^, we ascribed this effect to hydrogen, which diffuses into the silicon bulk during the firing step.

Through prolonged illumination at elevated temperatures (e.g., at 140 °C and 1 sun for 5 h) an improvement of the energy conversion efficiency by up to 0.2%_abs_ due to an absolute increase of both the open-circuit voltage and the short-circuit current density is possible. At the same time, a partial permanent deactivation of the responsible defect takes places, though degradation can still occur after regeneration with half the degradation extent as observed before permanent deactivation. Stable solar cell efficiencies above 22% were measured after regeneration, meaning that within the uncertainty range of the efficiency measurement, the developed cells on Ga-doped Cz-Si can be classified “long-term stable” after regeneration.

## Supplementary Information

Supplementary Figures.

## Data Availability

All data generated or analysed during this study are included in this published article.

## References

[1] Schmidt J (2004). Light-induced degradation in crystalline silicon solar cells. Solid State Phenom..

[2] Kersten F (2017). System performance loss due to LeTID. Energy Procedia.

[3] Krauss K, Fertig F, Menzel D, Rein S (2015). Light-induced degradation of silicon solar cells with aluminiumoxide passivated rear side. Energy Procedia.

[4] Ramspeck, K. *et al.* Light induced degradation of rear passivated mc-Si solar cells. in *Proceedings of the 27th EU PVSEC, Frankfurt, Germany,* 861–865 (2012).

[5] Bredemeier, D., Walter, D. C. & Schmidt, J. Lifetime degradation in multicrystalline silicon under illumination at elevated temperatures: Indications for the involvement of hydrogen. in *AIP Conference Proceedings, Lausanne, Switzerland,* 130001 (2018).

[6] Nakayashiki K (2016). Engineering solutions and root-cause analysis for light-induced degradation in p-type multicrystalline silicon PERC modules. IEEE J. Photovolt..

[7] Chan CE (2016). Rapid stabilization of high-performance multicrystalline P-type silicon PERC cells. IEEE J. Photovolt..

[8] Graf, A., Herguth, A. & Hahn, G. Determination of BO-LID and LeTID related activation energies in Cz-Si and FZ-Si using constant injection conditions. in *AIP Conference Proceedings, Leuven, Belgium,* 140003 (2019).

[9] Chen D (2017). Evidence of an identical firing-activated carrier-induced defect in monocrystalline and multicrystalline silicon. Sol. Energy Mater. Sol. Cells.

[10] Fertig F (2017). Mass production of p-type Cz silicon solar cells approaching average stable conversion efficiencies of 22 %. Energy Procedia.

[11] Niewelt T (2018). Understanding the light-induced degradation at elevated temperatures: Similarities between multicrystalline and floatzone p-type silicon. Prog. Photovolt. Res. Appl..

[12] Sperber D, Herguth A, Hahn G (2017). A 3-state defect model for light-induced degradation in boron-doped float-zone silicon. Phys. Status Solidi RRL.

[13] Chen D (2018). Hydrogen induced degradation: A possible mechanism for light- and elevated temperature- induced degradation in n-type silicon. Sol. Energy Mater. Sol. Cells.

[14] Grant NE (2020). Lifetime instabilities in gallium doped monocrystalline PERC silicon solar cells. Sol. Energy Mater. Sol. Cells.

[15] Grant NE (2021). Gallium-doped silicon for high-efficiency commercial passivated emitter and rear solar cells. Sol. RRL.

[16] Vicari Stefani B (2021). Stability study of silicon heterojunction solar cells fabricated with gallium- and boron-doped silicon wafers. Sol. RRL.

[17] Chen C, Wang H, Wang J, Lv J, Yang H (2022). Performance degradation of commercial Ga-doped passivated emitter and rear cell solar modules in the field. Progress Photovolt..

[18] Kwapil, W., Dalke, J., Niewelt, T. & Schubert, M. C. LeTID and (extended) BO-related degradation and regeneration in B- and Ga-doped monocrystalline silicon during dark and illuminated anneals. in *Proceedings of the 37th EU PVSEC, WIP, Munich,* 152–155 (2020).

[19] Kwapil W, Dalke J, Post R, Niewelt T (2021). Influence of dopant elements on degradation phenomena in B- and Ga-doped czochralski-grown silicon. Sol. RRL.

[20] Winter M, Walter D, Schmidt J (2021). Carrier lifetime degradation and regeneration in gallium- and boron-doped monocrystalline silicon materials. IEEE J. Photovolt..

[21] Lin D, Hu Z, Song L, Yang D, Yu X (2021). Investigation on the light and elevated temperature induced degradation of gallium-doped Cz-Si. Sol. Energy.

[22] Brendel, R. *et al.* Screening carrier selective contact combinations for novel crystalline Si cell structures. in *Proceedings of the 35th EU PVSEC, Brussels, Belgium,* 39–46 (2018).

[23] Min, B. *et al.* Approaching 23% with p-type back junction solar cells featuring screen-printed Al front grid and passivating rear contacts. in *Proceedings of the 38th EU PVSEC, WIP, Munich* (2021).

[24] Schmidt J, Cuevas A, Rein S, Glunz SW (2001). Impact of light-induced recombination centres on the current-voltage characteristic of czochralski silicon solar cells. Progress Photovolt..

[25] Sinton, R. A. & Cuevas, A. A quasi-steady-state open-ciruit voltage method for solar cell characterizsation. in *Proceedings of the 16th EU PVSEC, Glasgow, UK,* 1152–1155 (2000).

[26] Wolf M, Rauschenbach H (1963). Series resistance effects on solar cell measurements. Adv. Energy Conversion..

[27] Sperber D, Herguth A, Hahn G (2018). On improved passivation stability on highly-doped crystalline silicon and the long-term stability of regenerated Cz-Si. Sol. Energy Mater. Sol. Cells.

[28] Winter M, Bordihn S, Peibst R, Brendel R, Schmidt J (2020). Degradation and regeneration of n+ -doped poly-Si surface passivation on p-type and n-type Cz-Si under illumination and dark annealing. IEEE J. Photovolt..

[29] Maischner F (2022). LeTID mitigation via an adapted firing process in p-type PERC cells from SMART cast-monocrystalline, Czochralski and high-performance multicrystalline silicon. Progress Photovolt..

[30] Song L (2021). Performance improvement of gallium-doped passivated emitter and rear cells by two-step bias application. Sol. RRL.

