# Supplementary material for: Light and elevated temperature induced degradation and recovery of gallium-doped Czochralski-silicon solar cells
Source: Sci Rep. 2022 May 16;12:8089. doi: 10.1038/s41598-022-11831-3 (PMC9110720; doi:10.1038/s41598-022-11831-3)
Supplement: Supplementary file 1 — Supplementary Figures. [file 41598_2022_11831_MOESM1_ESM.pdf]

Light and elevated temperature induced degradation and recovery of gallium-doped Czochralski-silicon solar cells

Michael Winter[1,2,*], Dominic C. Walter[1], Byungsul Min[1], Robby Peibst[1], Rolf Brendel[1,2] & Jan Schmidt[1,2]

[1]Institute for Solar Energy Research Hamelin (ISFH), Am Ohrberg 1, 31860 Emmerthal, Germany.

[2]Department of Solar Energy, Institute of Solid-State Physics, Leibniz University Hannover, Appelstr. 2, 30167 Hannover, Germany.

*Corresponding author: m.winter@isfh.de (M. Winter).

Temperature-dependent light-induced degradation

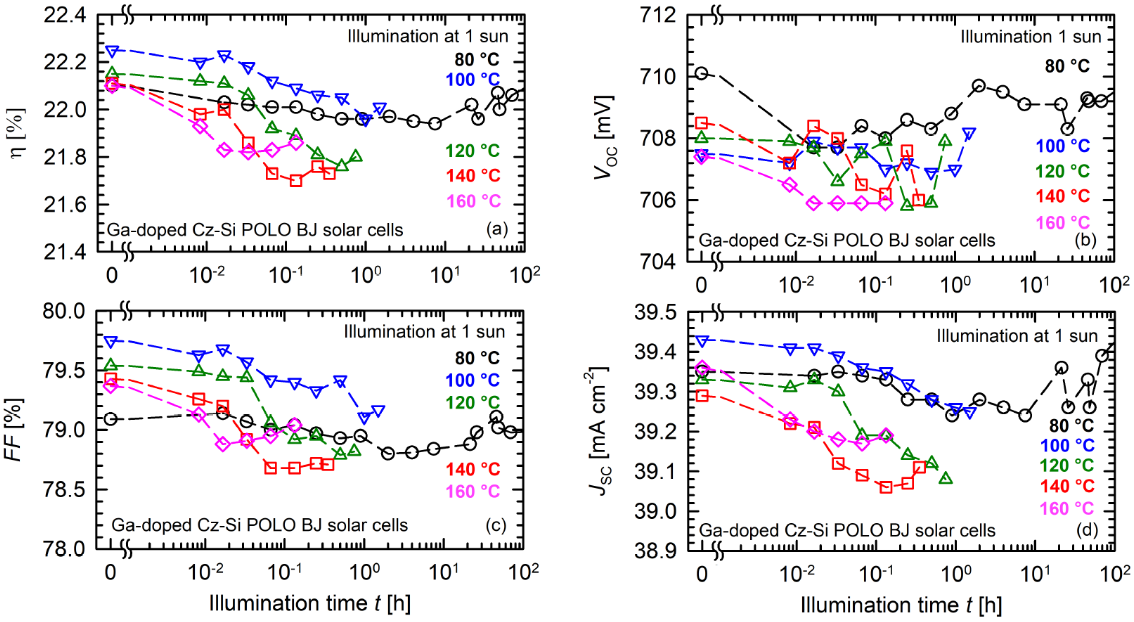

Supplementary Figure S1. Temperature-dependent light-induced degradation of Ga-doped Cz-Si POLO BJ solar cells at 1 sun light intensity and temperatures ranging from 80 to 160 °C. Shown are the changes in the measured cell parameters (a) energy conversion efficiency η, (b) open-circuit voltage V_{OC}, (c) fill factor FF, and (d) short-circuit current density J_{SC} versus the illumination time t. The discussion of the results has taken place in Fig. 3.

Permanent defect deactivation

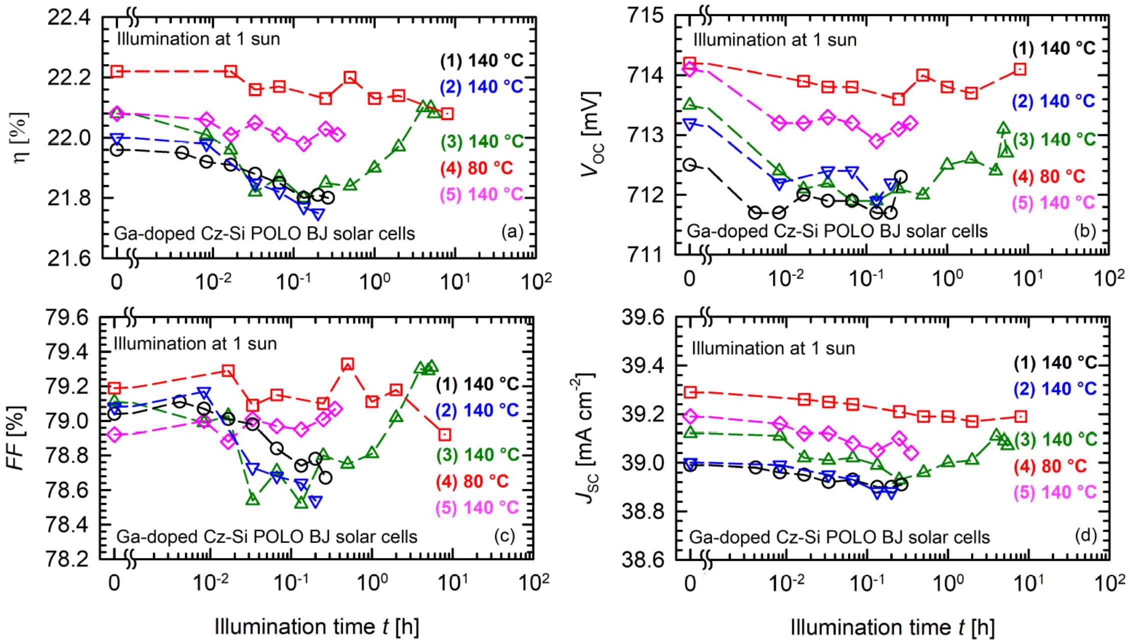

Supplementary Figure S2. Reversibility of a Ga-doped Cz-Si POLO BJ solar cell through consecutive activation (140°C, 1 sun, 12 min)/deactivation (44 °C, 0.5 suns, 15 min) cycles (1─3), regeneration through prolonged illumination at elevated temperatures (140°C, 1 sun, 5 h) (3), and test of the stability of the regeneration at 80 and 140 °C (4,5). Shown are the measured cell parameters (a) energy conversion efficiency η, (b) open-circuit voltage V_{OC}, (c) fill factor FF, and (d) short-circuit current density J_{SC}. The results have been discussed in Fig. 6.
